# A review of the impact of isolated antiphospholipid antibody positivity on pregnancy outcomes and clinical management strategies

**DOI:** 10.3389/fimmu.2026.1812670

**Published:** 2026-05-01

**Authors:** Xiangrui Wang, Shenglong Ye, Xueqing Zhao, Yongqing Wang

**Affiliations:** 1 Department of Obstetrics and Gynecology, Peking University Third Hospital, Beijing, China; 2 National Clinical Research Center for Obstetrics and Gynecology (Peking University Third Hospital), Beijing, China; 3 State Key Laboratory of Female Fertility Promotion, Department of Obstetrics and Gynecology Peking University Third Hospital, Beijing, China; 4 Key Laboratory of Assisted Reproduction (Peking University), Ministry of Education, Beijing, China; 5 Beijing Key Laboratory of Reproductive Endocrinology and Assisted Reproductive Technology, Beijing, China; 6 Department of Obstetrics and Gynaecology, Peking University People’s Hospital, Beijing, China

**Keywords:** adverse pregnancy outcome, antiphospholipid syndrome, isolated antiphospholipid antibody positivity, risk stratification, treatment

## Abstract

**Objective:**

This review summarizes the current evidence on the impact of isolated antiphospholipid antibody positivity (IAAP) on pregnancy outcomes. It analyzes risk differentials across antibody subtypes, synthesizes the underlying pathophysiological mechanisms, and reviews existing treatment strategies to inform clinical practice.

**Methods:**

A comprehensive review was conducted. Data were retrieved and analyzed from relevant observational, cohort, and randomized controlled trials, both domestically and internationally, focusing on clinical outcomes, risk stratification, pathogenic mechanisms, and the management of IAAP.

**Results:**

Positive antiphospholipid antibodies alone constitute a risk factor for adverse pregnancy outcomes. The pathological mechanisms linking IAAP to adverse outcomes involve multiple processes, including thrombosis, trophoblast dysfunction, and complement activation. Clinical management should involve risk stratification based on antibody profiles. For high-risk patients, a combination therapy of low-molecular-weight heparin and aspirin is recommended, with hydroxychloroquine as a potential adjuvant. Standardization of antibody testing and optimal timing for dynamic monitoring remain areas needing improvement.

**Conclusion and future perspectives:**

IAAP is associated with an increased risk of adverse pregnancy outcomes. Further research is needed to determine whether the presence of these antibodies may contribute to the development of autoimmune disease. Future research should focus on developing precise risk-stratification models that integrate antibody type, titer, and clinical features. Prospective studies are warranted to establish individualized monitoring and intervention protocols, ultimately contributing to an evidence-based clinical consensus.

## 1 Introduction

Antiphospholipid syndrome (APS) is an autoimmune disorder characterized by the persistent presence of antiphospholipid antibodies (aPLs) in the circulation as a laboratory criterion, and by pathological pregnancy or thromboembolism as clinical manifestations (1). Clear clinical guidelines for the diagnosis and treatment of APS have been established (2). However, in clinical practice, there are still some cases where patients have no history of pathological pregnancy and are found to have isolated antiphospholipid antibody positivity (IAAP) during incidental serological testing. It remains unclear whether IAAP constitutes a risk factor for pathological pregnancy, similar to APS; some studies suggest that IAAP is associated with adverse pregnancy outcomes (APOs) (3). In current clinical practice, there is a lack of effective evidence-based medical evidence regarding whether IAAP requires treatment and how to determine treatment strategies; furthermore, the prognosis regarding whether these pregnant will develop autoimmune diseases remains unclear. Therefore, the diagnosis, treatment, and management of this population present significant challenges.

Currently, there are no unified standards for pregnancy outcomes, antenatal management, or treatment in the IAAP population, nor are there studies on the dynamic monitoring of maternal autoimmune disease progression. Consequently, effective risk prediction models cannot be established, and clinical decision-making has long relied on empirical judgment, making it difficult to achieve precision diagnosis and treatment.

Against these background, this review aims to summarize current evidence regarding the impact of IAAP on pregnancy outcomes. We focus on demonstrating the risk stratification value of various aPLs subtypes (including non-standard aPLs), exploring their potential pathophysiological mechanisms, and evaluating existing management strategies. Finally, we propose future research directions: standardizing the definition and testing criteria for IAAP, conducting multicenter prospective studies, and developing dynamic multidimensional risk stratification models to guide evidence-based, individualized clinical diagnosis and treatment.

## 2 Antiphospholipid antibodies

APLs constitute a heterogeneous group of autoantibodies targeting phospholipids or phospholipid-binding proteins. The presence of these antibodies is closely associated with arterial and venous thrombosis and a spectrum of obstetric complications, establishing them as the core serological markers of APS. Immunochemically, aPLs exhibit target diversity, recognizing not only free anionic phospholipids (e.g., cardiolipin) but also complexes formed between phospholipids and plasma proteins (e.g., β2-glycoprotein I-phospholipid complexes). Current international diagnostic criteria rely on three classical antibody types: lupus anticoagulant (LAC), IgG/IgM anticardiolipin antibodies (aCL), and IgG/IgM anti-β2 glycoprotein I antibodies (aβ2GPI) (4, 5). Furthermore, studies indicate that non-criteria antibodies, such as anti-phosphatidylserine/prothrombin (aPS/PT), anti-phosphatidylethanolamine (aPE), anti-annexin A2/A5, and anti-phosphatidylinositol (aPI) antibodies, which target other phospholipids or phospholipid-binding proteins, are also implicated in the pathophysiology of APS (6).

The three standard antibodies are directly involved in the pathogenesis of APS. By recognizing phospholipids or phospholipid-binding proteins, they activate endothelial cells, platelets, and the complement system, thereby promoting thrombosis and pregnancy complications. Extensive studies have confirmed that persistent positivity for standard antibodies is significantly associated with thrombotic events (venous, arterial, or microvascular) and APOs, such as recurrent miscarriage and pre-eclampsia. Moreover, their detection methods have been standardized in clinical practice. International guidelines have established protocols for these assays, ensuring reproducibility and comparability of results (7). In contrast, non-criterion antibodies (e.g., anti-phosphatidylserine/prothrombin antibody, aPS/PT), while potentially increasing diagnostic sensitivity, suffer from inconsistent clinical interpretation and a lack of standardized detection methods, leading to variability in results (8). The clinical relevance and assay robustness of the standard antibodies, however, have been validated in large-scale cohorts (9).

## 3 Definition and diagnostic criteria for antiphospholipid syndrome

### 3.1 Definition

APS is an autoimmune disorder characterized by the persistent presence of circulating antiphospholipid antibodies. It manifests with various clinical phenotypes, including thrombosis in the venous, arterial, or microvascular systems, pathological pregnancy events, and other microvascular complications (1). Pathological pregnancy events encompass unexplained recurrent miscarriage and a spectrum of obstetric complications in late pregnancy, such as pre-eclampsia, preterm birth, placental insufficiency-related fetal growth restriction, and fetal death. Among these, recurrent miscarriage is the most frequently observed.

### 3.2 Diagnostic criteria

The diagnosis of APS requires meeting at least one clinical criterion and one laboratory criterion concurrently. Clinical criteria encompass vascular thrombosis, adverse pregnancy outcomes, and thrombocytopenia. Laboratory criteria are based on the persistent positivity of specific antibodies, including lupus anticoagulant (LA) positivity or medium-to-high titers of IgG/IgM anticardiolipin (aCL) and/or anti-β2 glycoprotein I (aβ2GPI) antibodies. This positivity must be confirmed on two or more occasions, with at least 12 weeks between them (**Table 1**) (2).

**Table 1:** Classification criteria for obstetric antiphospholipid syndrome (OAPS) according to the sydney criteria.

Clinical criteria	Laboratory criteria
At least one of the following criteria must be met: 1. One or more unexplained deaths of a morphologically normal fetus at or beyond 10 weeks of gestation, confirmed by ultrasonography or direct fetal examination. 2. One or more premature births of a morphologically normal neonate before 34 weeks of gestation due to: eclampsia or severe pre-eclampsia; recognized features of placental insufficiency.^a^ 3. Three or more consecutive, unexplained spontaneous abortions before 10 weeks of gestation, with maternal anatomical, hormonal, and both parental chromosomal factors having been excluded.	At least one of the following laboratory criteria must be met, with positivity confirmed on two or more occasions at least 12 weeks apart: 1. Presence of lupus anticoagulant (LA) in plasma. 2. Medium- or high-titer positivity (>40 GPL or MPL units, or above the 99th percentile) of IgG or IgM anticardiolipin antibodies (aCL) in serum or plasma. 3. Titer of IgG or IgM anti-β2 glycoprotein I antibodies (aβ2GPI) above the 99th percentile in serum or plasma.
Placental insufficiency features include: 1. Abnormal fetal monitoring (e.g., non-reactive nonstress test indicating fetal hypoxia); 2. Doppler flow abnormalities (e.g., absence of end-diastolic flow in umbilical artery); 3. Oligohydramnios; 4. Birth weight below the 10th percentile for gestational age. Diagnostic criteria require fulfillment of at least one clinical criterion and one laboratory criterion.	

LA, lupus anticoagulant; aCL, anti-cardiolipin antibodies; anti-β2GPI,anti-β2 glycoprotein I antibodies.

APS can be classified into thrombotic APS (TAPS) and obstetric APS (OAPS) (10, 11). TAPS primarily manifests as thrombus formation in arteries, veins, and microvessels, though the underlying thrombotic mechanisms remain unclear. OAPS refers to APS presenting with pathological pregnancy events. These events include unexplained recurrent miscarriage, fetal death, and late obstetric manifestations such as preeclampsia, preterm birth, or fetal growth restriction associated with placental dysfunction (12). Clinically, some patients exhibit features and laboratory criteria suggestive of OAPS but do not strictly meet the diagnostic criteria for OAPS. These cases are termed non-criteria obstetric antiphospholipid syndrome (NC-OAPS). Diagnostic standards for NC-OAPS lack global consensus, and varying criteria are often used across studies. Criteria include specific APS clinical manifestations and non-typical laboratory findings (two positive aPLs tests with an interval of less than 12 weeks; IgG/IgM aCL and/or anti-β2GPIAb levels of 20–39 GPL/MPL, or titers in the 95th–99th percentile) or typical laboratory findings with atypical clinical manifestations (two consecutive unexplained miscarriages; or three or more non-consecutive unexplained miscarriages; or late-onset preeclampsia; or placental hematoma, placental abruption, late preterm delivery), a diagnosis of NC-OAPS can be made (13).

In the pathogenesis of OAPS, antiphospholipid antibodies (aPLs) contribute to placental injury and pregnancy failure through multiple pathways and targets. The underlying mechanisms can be categorized into three principal aspects. First, aPLs directly impair trophoblast function by inhibiting their proliferation, migration, invasion, and differentiation. This leads to defective placentation, insufficient spiral artery remodeling, and consequent placental insufficiency (12). Second, aPLs extensively activate vascular endothelial cells, platelets, and the complement system (particularly the classical pathway) (10). This activation generates potent effectors, such as C5a, which in turn stimulate neutrophils to release neutrophil extracellular traps (NETs) and promote an inflammatory cytokine storm involving tumor necrosis factor-α (TNF-α), interleukin-6 (IL-6), and others. Consequently, a prothrombotic and proinflammatory microenvironment is established at the maternal-fetal interface, leading to placental microthrombosis and inflammatory injury. Third, aPLs disrupt local immune homeostasis. Decidual tissues from OAPS patients often exhibit abnormal infiltration of immune cells, including macrophages. The interaction between these cells and aPLs may further amplify the inflammatory response, aggravating tissue injury (10).

## 4 Isolated antiphospholipid antibody positivity and adverse pregnancy outcomes

IAAP is defined by the persistent presence of one or more antiphospholipid antibodies (aPLs) in the serum on two or more occasions at least 12 weeks apart, in the absence of any clinical symptoms of APS. This definition specifically excludes transient aPLs positivity secondary to other conditions, such as autoimmune diseases (e.g., systemic lupus erythematosus, SLE), infections, malignancies, or certain medications. This serological profile is increasingly being identified in clinical practice. However, standardized guidelines for the clinical management and treatment of individuals with IAAP are currently lacking.

All clinical studies included in this review enrolled participants who were positive for aPLs (including non-criteria antibodies related to APS), without a definite history of pathological pregnancy, thrombotic events, or other relevant clinical manifestations, and did not meet the diagnostic criteria for APS. Therefore, these participants were strictly defined as the population with IAAP.

In clinical practice, IAAP is most often identified in two scenarios. The first is its incidental discovery during routine health assessments or laboratory workups for unrelated conditions. Others are identified during screening for causes of miscarriage due to other factors such as embryonic chromosomal abnormalities or uterine malformations. Furthermore, IAAP may be identified during the workup of patients with unexpectedly prolonged activated partial thromboplastin time (APTT) or a false-positive syphilis serological test (14). Numerous studies have demonstrated that the incidence of adverse pregnancy outcomes (APOs)-such as miscarriage, preterm birth, and placental insufficiency-is significantly higher among aPLs-positive pregnant individuals compared to their aPLs-negative counterparts. For instance, in a cohort of 55 pregnant women with complete aPLs data, 50% of pregnancies with an adverse outcome were aPLs-positive, whereas only 4% of pregnancies without an adverse outcome tested positive (p=0.023) (15). This evidence underscores the heightened risk of APOs faced by individuals with IAAP and highlights the pressing clinical need for evidence-based intervention strategies (16).

A multicenter study by Fredi et al. (2018), involving 283 aPLs-positive pregnancies, reported an APO rate of 17.9% in patients with IAAP. This rate was comparable to the 17.7% observed in those meeting the full clinical criteria for obstetric APS. This finding suggests that aPLs positivity itself constitutes a significant risk factor for pregnancy complications, even in the absence of typical clinical manifestations (17). Another study in 2021, which included 708 women with a history of pathological pregnancy (case group) and 602 women with normal obstetric history (control group), found that the positive rates for individual antibodies (LA, aCL, aβ_2_GPI), for any combination of two or three antibodies, and the overall aPLs positivity were all significantly higher in the case group. The overall aPLs positivity was 14.69% in cases versus 0.66% in controls (P<0.05). Within the case group, the incidence of preterm birth was significantly higher among aPLs-positive women (50.97%) compared to aPLs-negative women (21.52%, P<0.05), indicating a close association between aPLs positivity and increased preterm birth risk (18).

Furthermore, Martínez-Zamora et al. found that recurrent pregnancy loss patients who were aPLs-positive had a significantly elevated long-term thrombotic risk (19.3%). This risk was markedly amplified when traditional cardiovascular risk factors such as hypertension and hyperlipidemia were present, with the incidence of thrombotic events being 42.8 times higher. This indicates that aPLs positivity is an independent risk factor for future thrombosis in patients with a history of recurrent pregnancy loss (19).

## 5 Antiphospholipid antibody subtypes and risk stratification

The combination and titer of different aPLs subtypes significantly influence the risk level for APOs. Positivity for lupus anticoagulant (LA) serves as a core high-risk marker, as its presence alone is significantly associated with pregnancy complications (20). Multivariable regression analysis has further quantified these risks: LA positivity increases the risk of APOs by 7-fold (OR = 7.0, 95% CI 3.4–14.4), whereas positivity for anti-β2GPI domain 1 antibodies (aD1) elevates the risk by 12-fold (OR = 12.1, 95% CI 3.0–48.5) (6). When LA, aD1, and anti-phosphatidylserine/prothrombin (aPS/PT) IgG are all positive concurrently, the incidence of APOs rises dramatically to 47.6%, far exceeding that associated with other antibody profiles (6). In contrast, individuals with isolated aCL positivity or low-titer anti-β2GPI antibodies carry a comparatively lower risk (16).

### 5.1 Antibody subtypes

#### 5.1.1 Criterion antibodies

##### 5.1.1.1 Lupus anticoagulant

LA is a strong predictor of APOs, with its risk being particularly pronounced for late pregnancy loss. Even in isolation, its presence indicates high risk, and it is the most frequently detected aPLs type in clinical practice.

Opatrny et al. suggested that among women without autoimmune disease, IAAP is associated with recurrent pregnancy loss, with the strength of association varying by antibody type, titer, and timing of loss. LA demonstrated the strongest association with late recurrent loss (<24 weeks) (OR 7.79, 95% CI 2.30-26.45) (20). Consistent with its high-risk profile, Liu et al. (2025) identified four distinct clinical subgroups in an analysis of 528 pregnancies in SLE patients. The subgroup characterized by LA positivity and high clinical disease activity had the highest incidence of APOs (75.6%), whereas patients with non-LA aPLs had a lower risk (21).

Furthermore, Qi et al. (2022) conducted a prospective cohort analysis of 383 aPLs-positive patients and identified four clinical phenotypes: Cluster 1 (patients with SLE and non-criteria manifestations), Cluster 2 (patients with multiple cardiovascular risk factors), Cluster 3 (women with predominant obstetric events), and Cluster 4 (patients with isolated LA positivity). The long-term prognosis and thrombotic risk for patients with isolated LA positivity were similar to those in Clusters 1 and 2, indicating that isolated LA positivity defines a high-risk subgroup even in the absence of formal APS diagnostic criteria (22).

Regarding prevalence, Khefacha et al. (2022) reported that among women with OAPS, LA was the most common aPLs (7.54%), followed by aCL (3.77%) and anti-β_2_GPI (1.88%) (23). Martins et al., in a study of 1179 patients suspected of having APS, found an overall aPLs positivity rate of 17.9%. LA was again the most prevalent type (11.8%), followed by anti-β_2_GPI IgM (3.4%) and anti-β_2_GPI IgG (3.0%). The study also noted a higher prevalence of LA positivity among inpatients (21.4%) compared to outpatients (16.0%) (24).

##### 5.1.1.2 Anticardiolipin antibody

Anticardiolipin antibodies (aCL), particularly IgG isotypes at medium-to-high titers, are established risk factors for both early and late recurrent pregnancy loss. Evidence also suggests an association between low-titer aCL and APOs.

Pires Da Rosa et al. (2021) reported that low-titer aCL (11–40 GPL/MPL) is associated with pregnancy complications. Although the incidence of APOs (e.g., miscarriage, preterm birth) in this group is lower than in those with medium-to-high titers, it remains significantly higher than in antibody-negative individuals (25). A meta-analysis by Opatrny et al. (2006) confirmed that IgG aCL is associated with both early (<13 weeks) and late recurrent loss (OR 3.56 and 3.57, respectively). This association was strengthened for medium-to-high titer IgG aCL (OR 4.68, 95% CI 2.96-7.40). In contrast, IgM aCL showed a significant association only with late recurrent loss (OR 5.61, 95% CI 1.26-25.03), while anti-β2GPI antibodies were not significantly associated with early recurrent loss (OR 2.12, 95% CI 0.69-6.53) (20).

Yetman and Kutteh (1996), in a study of 866 women with recurrent pregnancy loss, found that 17.3% were positive for aCL. An additional 10.1% of women were aCL-negative but positive for other antiphospholipid antibodies (e.g., anti-phosphatidylinositol, -glycerol, -serine, -ethanolamine) (26).

Research by Do Prado et al. indicates that medium-to-high titer aCL is associated with pre-eclampsia, with an OR of 2.86 (95% CI 1.37–5.98) for pre-eclampsia and a substantially higher OR of 11.15 (95% CI 2.66–46.75) for severe pre-eclampsia, suggesting a stronger link with the severe form of the disease (27). This is corroborated by findings of a higher detection rate of aCL among patients with severe pre-eclampsia (28).

##### 5.1.1.3 Anti-β2 glycoprotein I antibody

Anti-β2-glycoprotein I antibodies are considered pivotal in the pathogenesis of APS. Research highlights the distinct roles of different antibody isotypes. Nonobe M et al. (2025) identified IgG anti-β2GPI as an independent risk factor for intrauterine fetal death (OR 1.05, 95% CI: 1.01–1.10, p = 0.008), with a predictive performance of 0.80 in models incorporating prior history of stillbirth (29).

In contrast, Song Y et al. (2017) focused on the IgM isotype in APS patients with recurrent pregnancy loss. IgM anti-β2GPI was the predominant antibody (detected in all 123 patients, with only 10.6% co-positive for aCL IgM), and the magnitude of its titer reduction during treatment strongly correlated with pregnancy outcome. The live birth group showed a more significant titer decrease (from 56.8 ± 49.0 RU/ml pre-treatment to 24.1 ± 23.1 RU/ml in early pregnancy, P < 0.05) compared to the miscarriage group (52.8 ± 30.7 RU/ml to 33.9 ± 24.7 RU/ml, P < 0.001). Patients with inadequate titer reduction had lower success rates, indicating that dynamic titer decline is a key indicator of treatment efficacy (30).

In line with its broader biomarker role, Mattia et al. (2014) found IgA anti-β2GPI in 50% of primary APS patients and 10.6% of seronegative APS patients. Its titer was significantly higher in thrombotic patients (P = 0.034), suggesting it may serve as a potential marker in IAAP, associated with both thrombotic risk and adverse pregnancy outcomes (31).

#### 5.1.2 Non-criterion antibodies

##### 5.1.2.1 Anti-phosphatidylserine/prothrombin antibody

Among non-criteria antiphospholipid antibodies, aPS/PT is among the most extensively studied. Accumulating evidence demonstrates its clear clinical relevance and close association with both APS and lupus anticoagulant (LA) (32).

Specifically, Xiang J et al. (2024) reported a significant association between aPS/PT and adverse pregnancy outcomes. Both aPS/PT IgG and IgM were identified as independent risk factors for pregnancy loss, with odds ratios (ORs) of 1.055 (95% confidence interval [CI]: 1.009–1.103, p = 0.017) and 1.041 (95% CI: 1.015–1.067, p = 0.002), respectively. Notably, the IgM isotype showed a positive linear relationship with miscarriage risk (33).

##### 5.1.2.2 Anti-β2 glycoprotein I domain I antibody

In the PROMISSE prospective cohort study, Moyle, K. A. et al. (2025) identified aD1 as one of the strongest independent predictors of adverse pregnancy outcomes. It achieved an area under the receiver operating characteristic curve (AUC) of 0.734, with an adjusted odds ratio of 12.1 in multivariable analysis. The predictive significance of aD1 persisted even after adjusting for lupus anticoagulant (LA). The study further revealed a very strong association between aD1 and LA positivity (OR = 27.9 when co-positive with aPS/PT IgG), suggesting that aD1 may be the key pathogenic antibody underlying a positive LA test (6).

##### 5.1.2.3 Other non-criterion antibodies

Anti-Annexin V Antibody (aAnnexinV): Cross-sectional studies have reported a relatively high sensitivity (58.86%) but low specificity (55.81%) for aAnnexinV. Evidence for a specific association with APOs remains insufficient, suggesting its primary value may lie in assisting the identification of seronegative APS (32).

Anti-Phosphatidylethanolamine Antibody (aPE) and Anti-Phospholipid Mixture Antibody (APhL): Current research has not established a direct link between these antibodies and APOs. Although APhL IgG is associated with thrombotic risk (particularly arterial thrombosis), data on pregnancy outcomes are inadequate (32).

Anti-Phosphatidylserine Antibody (aPS IgG):Some studies suggest an association with thrombotic risk. However, as an independent predictor of pregnancy outcomes, the strength of evidence for aPS IgG is considerably weaker compared to aD1 and aPS/PT IgG (6).

### 5.2 Risk stratification

Current literature primarily stratifies risk based on the three standard antibody types (LA, aCL, anti-β2GPI), classifying profiles into triple, double, or single positivity based on the number of antibodies detected.

#### 5.2.1 Triple positivity

The “triple positive” profile (concurrent positivity for LA, aCL, and anti-β2GPI) is associated with higher clinical risks, including thrombosis and pregnancy complications (34, 35). This profile is associated with a significantly lower live birth rate than other serological profiles, although the findings require validation in larger cohort studies (36).

#### 5.2.2 Double positivity

Existing research has not established a significant statistical association between double positivity for the standard antibodies and APOs (17). However, studies on non-criterion antibodies indicate that double positivity for aD1 and aPS/PT IgG confers a higher risk of APOs than single positivity. This specific combination is also strongly correlated with LA positivity, which is itself a key predictor of adverse pregnancy outcomes (6).

#### 5.2.3 Persistent antibody positivity

High titers of aCL and anti-β2GPI are linked to increased clinical risk. Persistent, rather than transient, antibody positivity further elevates this risk (35, 37). The persistence of aPLs positivity (≥12 weeks) is a core diagnostic criterion for APS and is significantly associated with both APOs and thrombosis (38, 39). The link between antibody persistence and pregnancy complications (e.g., pre-eclampsia, fetal growth restriction) is particularly pronounced in SLE patients who are aPLs-positive (40).

#### 5.2.4 Non-criterion antibody positivity

Beyond the standard antibodies, other aPLs may influence pregnancy outcomes through unknown mechanisms, but the evidence remains limited (41, 42). Non-criterion aPLs (e.g., aPS/PT, aPE, anti-Annexin V) may enhance the diagnostic sensitivity for APS, particularly in identifying patients who meet clinical criteria but test negative for standard antibodies. Their independent predictive value warrants further investigation: studies confirm that aD1 and aPS/PT remain significantly associated with adverse pregnancy outcomes even after adjusting for LA (aD1 OR 12.1; aPS/PT OR 11.4) (25), yet they are not included in current diagnostic criteria (8, 43).

## 6 Proposed pathogenic mechanisms

**Figure 1:**
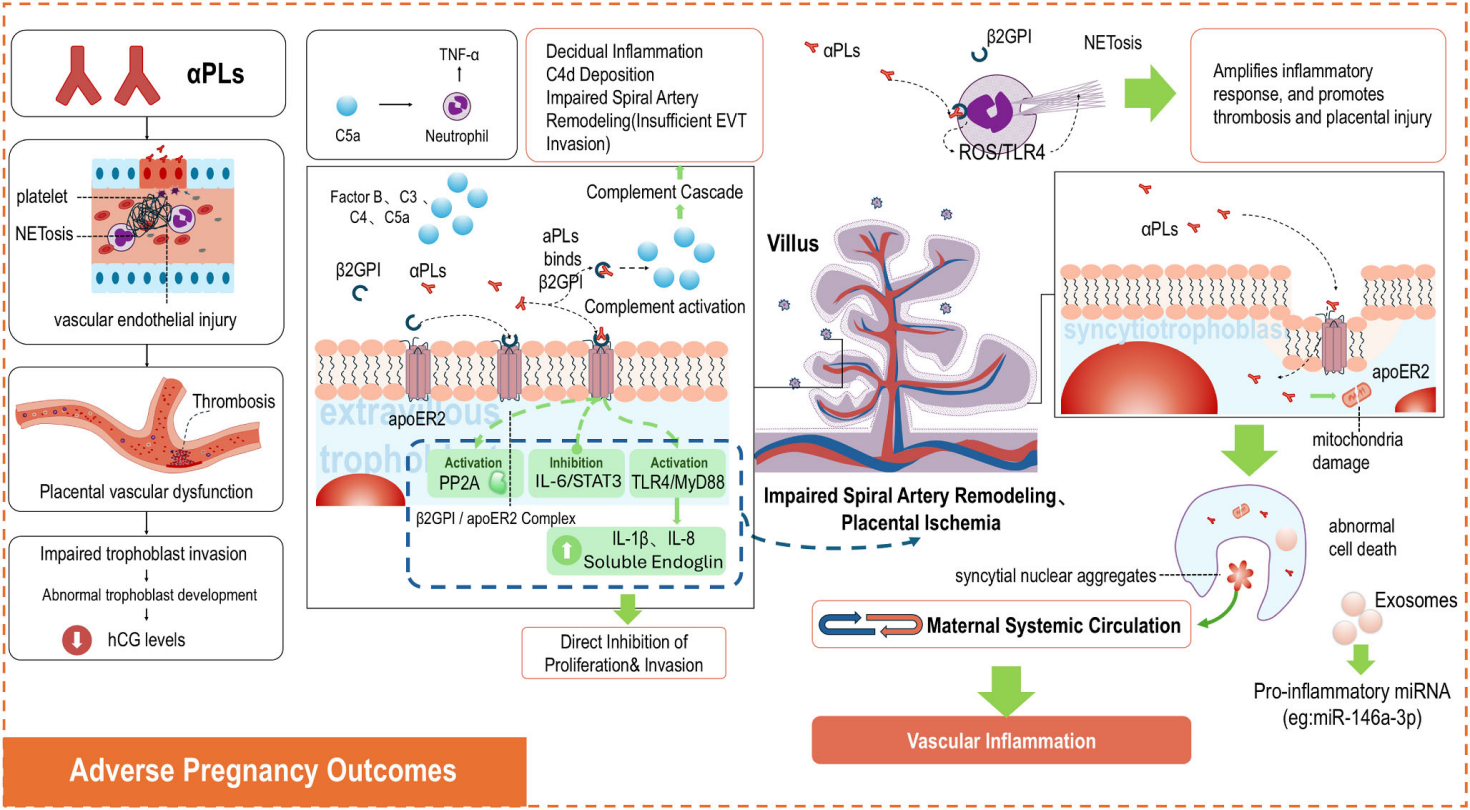
Antiphospholipid antibodies contribute to adverse pregnancy outcomes through multiple pathways, including inflammation, immune dysregulation, thrombosis, and impaired trophoblast function.

Antiphospholipid antibodies contribute to adverse pregnancy outcomes through multiple pathways, including inflammation, immune dysregulation, thrombosis, and impaired trophoblast function (**Figure 1**).

### 6.1 Placental microthrombosis and ischemic injury

Placental microthrombosis is considered a classic pathological feature of OAPS-related pregnancy complications. The “two-hit” model has been proposed to explain this process. The first hit involves aPLs-induced endothelial damage, which impairs vascular function and creates a pro-thrombotic state. The second hit, often triggered by an inflammatory stimulus or trauma, involves the binding of β2GPI to the damaged endothelium. This interaction is crucial for initiating localized thrombosis (12). Furthermore, aPLs binding to β2GPI can further activate endothelial cells and platelets, promoting microthrombus formation (44). The progressive occlusion of placental vessels by these microthrombi leads to focal ischemia and necrosis. This results in diminished trophoblast function, including impaired invasion and syncytiotrophoblast dysfunction, reduced secretion of human chorionic gonadotropin (hCG), and ultimately compromised placental function and pregnancy outcomes.

### 6.2 Complement system activation

aPLs, particularly anti-β2GPI antibodies, can cause placental injury by activating the complement system (C3, C4, factor B) (12). The aPLs/β2GPI complex primarily drives the classical complement pathway, leading to the formation of membrane attack complexes that cause direct cellular damage at the maternal-fetal interface (45). Complement activation can also occur via the factor B-mediated alternative pathway, further promoting membrane attack complex formation (46). The complement activation product C5a can amplify this injury by enhancing tumor necrosis factor-α (TNF-α) expression and stimulating neutrophil tissue factor production. This cascade further disrupts angiogenesis and compromises placental blood flow (47).

### 6.3 Neutrophil extracellular trap-mediated placental injury

Neutrophil extracellular traps are web-like structures released by neutrophils via a unique form of programmed cell death called NETosis, which functions to trap and kill pathogens (48). Under pathological conditions, NETs not only amplify inflammation but also promote thrombosis and placental injury (49). Anti-β2GPI antibodies can bind to β2GPI expressed on neutrophil surfaces, triggering NET release through reactive oxygen species (ROS) generation and a Toll-like receptor 4 (TLR4)-dependent pathway. Complement activation can indirectly enhance the interaction between neutrophils and β2GPI further augments NET release (50).

### 6.4 Trophoblast dysfunction

aPLs impair trophoblast function through two primary avenues. First, they directly inhibit the proliferation and invasion of extravillous trophoblasts. Second, they induce dysfunction via the low-density lipoprotein receptor apoER2. Binding of anti-β2GPI antibodies to the β2GPI/apoER2 complex activates protein phosphatase 2A (PP2A), contributing to maternal hypertension, proteinuria, and fetal growth restriction (12).

Specifically, aPLs inhibit extravillous trophoblast migration and invasion by blocking IL-6/STAT3 signaling via the ApoER2 receptor. Concurrently, they activate the TLR4/MyD88 pathway, promoting the release of inflammatory cytokines such as IL-1β and IL-8 and stimulating the secretion of anti-angiogenic factors, such as soluble endoglin. These alterations collectively lead to impaired spiral artery remodeling and placental ischemia.

Regarding syncytiotrophoblasts, aPLs are internalized via low-density lipoprotein receptor family members, causing mitochondrial damage and abnormal cell death. This process promotes the release of syncytial nuclear aggregates and other dangerous microparticles into the maternal circulation, activating systemic vascular inflammation. Furthermore, aPLs suppress human chorionic gonadotropin (hCG) secretion and increase the release of pro-inflammatory miRNAs (e.g., miR-146a-3p) within exosomes, thereby exacerbating placental dysfunction and systemic inflammation (44). The interplay of these mechanisms ultimately leads to severe pregnancy complications.

## 7 Current clinical management and treatment

IAAP warrants attention regarding adverse pregnancy outcomes and the long-term prognosis of autoimmune diseases.

Regarding treatment protocols, no authoritative guidelines based on high-level evidence currently exist domestically or internationally. Treatment choices rely heavily on individual physician experience and regional practices, resulting in significant inconsistencies. Specifically, treatment approaches range from simple clinical observation to low-dose aspirin (LDA) alone, to combination therapy with LDA and low-molecular-weight heparin (LMWH), or to the addition of immunomodulators like hydroxychloroquine (HCQ) on top of these regimens, reflecting substantial variation in protocol selection. Furthermore, safety concerns regarding anticoagulants (such as LDA and LMWH) during pregnancy further complicate clinical decision-making and reinforce conservative tendencies.

Regarding management, there is currently no systematic protocol for monitoring pregnant women amid fluctuating immune states. No standardized monitoring or assessment tools exist for either dynamically evaluating risks of APOs like placental dysfunction or tracking maternal disease progression—specifically thromboembolic events and long-term AID outcomes. Current clinical research on thromboembolic events and long-term AID outcomes after pregnancy termination in these patients is virtually nonexistent. Management relies heavily on individual physician experience, with variations in follow-up protocols, monitoring frequencies, and intervention thresholds among clinicians. This inconsistency hinders early risk identification and the implementation of standardized prevention strategies.

### 7.1 Anticoagulation and antiplatelet therapy

#### 7.1.1 Low molecular weight heparin + aspirin

Multiple studies indicate that patients with IAAP may benefit from anticoagulation regimens identical to those used for APS. Low-dose aspirin (LDA) combined with low-molecular-weight heparin (LMWH) is a first-line treatment regimen that effectively improves pregnancy outcomes. A Dutch review encompassing 11 studies (1,672 women) indicated that LDA combined with LMWH during pregnancy may increase the live birth rate in women with persistent aPLs positivity (51). Additionally, studies suggest this regimen generally reduces the miscarriage risk in aPLs-positive women (RR 0.48); the effect of LDA alone remains unclear (52). Some studies suggest clinical decisions should be guided by antibody profiles: high-risk patients (e.g., LA-positive, triple-positive) require anticoagulant therapy, while close monitoring may be more appropriate for isolated aCL-positive cases without complications (23, 24). For patients with adverse obstetric histories who do not meet APS diagnostic criteria, LDA or LDA combined with LMWH may be considered, though further evidence is needed (25). Current clinical LDA dosing typically ranges from 75–100 mg/day, administered from early pregnancy through delivery. LMWH prophylactic doses are generally 4000 IU/day, initiated in early pregnancy and often continued postpartum (53).

#### 7.1.2 Vitamin K antagonists

Vitamin K antagonists constitute the standard therapy for thrombotic antiphospholipid syndrome (54). For IAAP patients, long-term prognosis after pregnancy termination requires further assessment of their future thrombotic risk. In non-pregnant IAAP patients with elevated thrombotic risk, vitamin K antagonists may serve as a secondary prevention option. Studies by Hubben and McCrae (2023) and Legault indicate that VKAs represent the standard oral regimen for thrombotic event prevention, particularly in patients with prior thrombosis. Maintaining an International Normalized Ratio (INR) within the target range of 2.0–3.0 effectively prevents recurrence without requiring higher anticoagulation intensity. In contrast, direct oral anticoagulants (DOACs, such as rivaroxaban) demonstrated higher thrombotic recurrence rates in clinical trials, such as TRAPS, and are therefore not recommended for thrombotic APS patients with high-risk features (e.g., triple antibody positivity) (55, 56).

### 7.2 Hydroxychloroquine

Hydroxychloroquine (HCQ) has potential as an adjunct therapy to improve adverse pregnancy outcomes associated with antiphospholipid antibodies (aPLs). Tian et al. (2021) meta-analysis indicated that adding HCQ to standard treatment significantly increased live birth rates and reduced miscarriage risk, though its effect on preventing preterm birth or fetal growth restriction was not significant (57). A retrospective study by Chen et al. (2024) on patients with low-titer aPLs-positive recurrent miscarriage (RM) further supported the necessity of treatment, finding that appropriate interventions-particularly multi-drug regimens including HCQ-significantly reduced recurrent miscarriage rates and improved live birth rates (58). Ye et al. (2025) demonstrated that HCQ can prevent adverse pregnancy outcomes by inhibiting excessive autophagy in trophoblast cells induced by aPLs (59). Thus, HCQ may serve as an adjunctive therapeutic option to improve pregnancy outcomes in aPLs-positive patients, particularly those with adverse obstetric histories. However, its optimal patient population and long-term benefits require further validation through high-quality studies. Hydroxychloroquine is generally safe during pregnancy, with the most common adverse reactions being mild gastrointestinal symptoms and rash. Long-term use requires vigilance for dose-related retinal toxicity; a daily dose not exceeding 5 mg/kg is recommended, along with regular ophthalmic monitoring. Regarding pregnancy safety, substantial evidence indicates that doses below 400 mg/day do not increase the risk of fetal congenital malformations (60).

### 7.3 Conventional therapy combined with intravenous immunoglobulin

Intravenous immunoglobulin (IVIG) is a medication derived from the plasma of thousands of healthy blood donors, containing multiple antibodies capable of modulating immune responses (61). Originally used as replacement therapy for patients with immunodeficiency, IVIG’s anti-inflammatory and immunomodulatory effects have led to its approval for treating various autoimmune and inflammatory diseases, such as Kawasaki disease, immune thrombocytopenia, and Guillain-Barré syndrome (62). Regarding IVIG use in aPLs-positive patients, limited clinical research has explored its therapeutic potential. A systematic review and meta-analysis by Yuan et al. (2024) included 9 randomized controlled trials involving 366 high-risk aPLs-positive women to assess whether intravenous IVIG could improve live birth rates and neonatal outcomes in IAAP patients. Comparisons between intervention and control groups showed no significant differences in obstetric complications or neonatal outcomes. The IVIG group exhibited a higher preterm birth rate than the control group (OR = 2.05, I² = 46%, 95% CI [0.58-5.24]), but also demonstrated improved live birth rates (OR = 2.86, I² = 52%, 95% CI [1.04–7.90]), as it reduced the number of miscarriages (OR = 0.35, I² = 52%, 95% CI [0.13–0.96]). This suggests IVIG intervention may be a potentially effective approach for managing aPLs-positive pregnant women at high risk of miscarriage (63). Common adverse reactions to IVIG are mostly mild and transient, such as fever, headache, and nausea. Clinical application is generally safe and well-tolerated (64). Regarding its mechanism of action, IVIg may act by blocking Fc receptors or accelerating antibody clearance, though its efficacy remains to be fully established (65).

### 7.4 Biologics

For patients with autoimmune diseases (such as systemic lupus erythematosus and antiphospholipid syndrome) who exhibit poor response or intolerance to traditional immunosuppressive agents, biologics and small-molecule drugs have emerged as important alternative treatment options (66). Common biologics include monoclonal antibodies and tumor necrosis factor inhibitors, typically antibodies or antibody-based proteins that modulate immune responses by specifically targeting key molecules in the immune system (67). Treatment strategies and corresponding evidence for individuals with IAAP remain unclear. Case reports indicate that anti-CD38 monoclonal antibodies (daratumumab) can directly suppress sustained aPLs production by eliminating long-lived plasma cells. In refractory APS patients, this therapy demonstrated favorable safety and clinical tolerability, significantly reduced aPLs levels, and improved clinical symptoms (68). Future studies are needed to clarify the efficacy, safety, optimal timing, and long-term prognosis of biologics in this population, thereby providing evidence for precise clinical interventions.

### 7.5 Glucocorticoids

For refractory obstetric APS and SLE patients with positive aPLs who fail conventional anticoagulant therapy, glucocorticoids (GCs) may be considered. Their primary mechanism involves potent, broad-spectrum anti-inflammatory and immunosuppressive effects that can control the underlying immune inflammation in SLE (69). Additionally, studies suggest GCs’ immunomodulation may influence aPLs levels, though the precise mechanism remains unclear (70). Clinically, for refractory obstetric APS unresponsive to conventional anticoagulation, low-dose prednisone (≤10 mg/day in early pregnancy) or equivalent doses of other GCs may be added to the pre-pregnancy regimen of LDA and HCQ (13). However, these medications carry well-documented side effects, with long-term use increasing risks of metabolic disorders and infections (71). This compounds the inherent thrombotic risk in IAAP patients, further complicating treatment. Clinical application requires individualized assessment, and future research should explore the specific mechanisms by which GCs modulate the aPLs immune response to guide personalized dosing.

### 7.6 Other therapies

Beyond conventional anticoagulation and immunomodulation strategies, targeted therapies addressing specific pathological pathways hold research value. Studies indicate that phosphodiesterase-3 (PDE3) inhibitors, by elevating intracellular cAMP levels, inhibit aPLs-induced platelet activation in experimental models. Concurrently, restoring platelet homeostasis may be achievable by enhancing CD73-mediated adenosine production or employing A2AR agonists (72). Furthermore, targeting KLF2-a key transcription factor in neutrophil activation—is also considered a potential therapeutic avenue (50).

## 8 Discussion and outlook

### 8.1 Risk of adverse pregnancy outcomes associated with isolated anti-phospholipid antibody positivity

Whether IAAP constitutes an independent risk factor for APO remains controversial. Numerous studies indicate a significantly higher aPLs positivity rate in the APO group compared to the group without adverse outcomes, with IAAP showing a markedly higher incidence of adverse pregnancy outcomes than the antibody-negative group (15). However, the incidence of adverse outcomes varies considerably across different cohorts (73), necessitating further research to clarify the risk. This variability may stem from the complexity of the aPLs antibody profile, confounding comorbidities, and limitations in sample size. Future large-scale, multicenter prospective studies incorporating comprehensive antibody profiling data are needed. Detailed stratified analyses should clarify the risk intensity associated with different antibody characteristics, enabling the development of independent risk assessment models that account for confounding factors.

### 8.2 Standardization of aPLs testing and optimal monitoring duration

aPLs positivity requires the sustained presence of one or more aPLs in serum across at least two independent tests conducted at least 12 weeks apart. However, there is no consensus on the optimal testing interval. aPLs levels fluctuate during pregnancy, and their temporal relationship with APO remains unclear (74, 75). Most existing studies employ single-time-point testing, making it difficult to establish an effective early warning window. Inadequate standardization of aPLs testing kits significantly compromises the accuracy and comparability. LA detection exhibits pronounced reagent dependency, leading to significant differences in positive rates between different reagents (90% vs. 65%, p=0.05) (29, 76). Non-standardized antibody testing for aPLs lacks standardized protocols, resulting in poor comparability of quantitative results across platforms (29, 77) and limiting the development of risk-stratification models based on antibody profiles. Furthermore, given the substantial presence of IAAP-positive individuals in clinical settings, whether to incorporate this test into routine prenatal screening remains to be substantiated by further research. Future longitudinal studies are needed to clarify the dynamic changes of relevant antibodies. Time-dependent ROC analysis should determine optimal testing timepoints and combined monitoring strategies. International efforts to standardize and validate test kits, establish uniform testing protocols and reference ranges, and lay the foundation for antibody-based risk-stratification models are also essential.

### 8.3 Clinical management of IAAP individuals

Clinical management for IAAP individuals should span the entire pregnancy and include long-term follow-up, with a standardized long-term management system established. In obstetric management during pregnancy, the key lies in establishing risk stratification based on antibody positivity. Factors such as antibody combination patterns, titer levels, antibody specificity, and duration should be comprehensively considered to enhance monitoring for high-risk individuals. This includes determining indications for drug therapy, timing of intervention, and specific intervention measures. For low-to-moderate risk individuals, appropriate testing frequencies should be established while vigilantly monitoring for potential progression to high-risk status. Concurrently, efforts should advance reagent standardization and dynamic antibody-level research to clarify optimal monitoring time points. Further investigation is needed to determine whether aPLs testing should be incorporated into routine prenatal care for high-risk populations. Regarding long-term prognosis, the risk of IAAP progressing to AIDS remains unclear. Large-scale prospective studies are needed to conduct long-term post-pregnancy follow-up for these patients. Collaborative management with rheumatology departments is essential, involving regular assessment of antibody profiles, inflammatory markers, and thrombotic risk factors to identify predictors of long-term progression to AID. Exploring corresponding intervention strategies is crucial to safeguard the long-term health of the IAAP population.

### 8.4 Limitations of this review

Although this review provides a comprehensive overview, it has certain limitations. First, as a narrative review, it does not conduct a quantitative meta-analysis to synthesize the data and is therefore inevitably susceptible to selection bias and publication bias. Second, the included original studies exhibit significant heterogeneity in terms of antibody detection methods, testing platforms, and cut-off values, which complicates direct data comparison and limits the generalizability of clinical findings. Third, current evidence regarding non-standard antibodies and long-term autoimmune progression in IAAP patients primarily stems from relatively small observational cohorts rather than large-scale, multicenter randomized controlled trials. Consequently, the strength of clinical recommendations derived from these studies remains limited, underscoring the urgent need for further standardized, high-quality prospective studies to validate these conclusions.

## 9 Conclusion

Positive antiphospholipid antibodies alone constitute a risk factor for adverse pregnancy outcomes. Beyond these outcomes, close attention should be paid to the potential progression to true autoimmune diseases.

Future large-scale studies are needed to clarify their independent risk and to stratify antibodies. The optimal timing for dynamic monitoring of aPLs remains undetermined, and whether they should be incorporated into routine prenatal checkups remains a matter of ongoing research to establish consensus. Additionally, variations in antibody detection results across different reagents and platforms may impact the clinical implementation of risk stratification models. Large-scale standardized validation is needed to establish unified antibody testing standards.

The core direction of clinical management is establishing individualized strategies based on antibody characteristics. Future research should focus on refining risk stratification according to antibody profiles, titers, and specificities, thereby developing differentiated monitoring and intervention plans to achieve precision management. Ultimately, high-quality evidence should inform the development of unified clinical pathways and expert consensus to standardize management and improve pregnancy outcomes and long-term prognosis.
